# How Much Country Economy Influences ECC Profile in Serbian Children—A Macro-Level Factor Analysis

**DOI:** 10.3389/fpubh.2019.00285

**Published:** 2019-10-11

**Authors:** Dejan Markovic, Ivan Soldatovic, Rade Vukovic, Tamara Peric, Guglielmo Giuseppe Campus, Ana Vukovic

**Affiliations:** ^1^Department of Pediatric and Preventive Dentistry, School of Dental Medicine, University of Belgrade, Belgrade, Serbia; ^2^Department of Statistics and Bioinformatics, School of Medicine, University of Belgrade, Belgrade, Serbia; ^3^School of Medicine, University of Belgrade, Belgrade, Serbia; ^4^Mother and Child Healthcare Institute of Serbia Dr Vukan Cupic, Belgrade, Serbia; ^5^Department of Dentistry, Preventive and Pediatric Dentistry, University of Bern, Bern, Switzerland

**Keywords:** caries, child preschool, epidemiology, prevalence, universal coverage, economy

## Abstract

**Introduction:** Serbia has universal health coverage (UHC) for pediatric dental care and similar country distribution for dentists and physicians per 1,000 inhabitants. However, a high prevalence of early childhood caries (ECC) with wide variation across the country was observed in previous studies. This paper aimed to analyze the association between economic and healthcare country macro-level factors with ECC prevalence and treatment.

**Method:** The outcome variables were ECC prevalence and frequency of untreated ECC in 36- to 71-month-olds. Cross-sectional pathfinder survey on a nationally representative sample of children was conducted in order to obtain data. Independent variables included the following: gross domestic product (GDP), social and health care budget beneficiaries' expenditures, local self-government budget, unemployment rate, population density and density of physicians and dentists. Guided by the WHO's Basic Methods for Oral Health Surveys stratified cluster sample, 17 sites were randomly chosen to obtain adequate distribution of data regarding urban, peri-urban and rural areas in each analyzed statistical territorial unit. The variables were analyzed using the independent *t*-test or Mann–Whitney *U* test. A probability value of <0.05 was considered significant.

**Results:** The final sample included 864 children aged 36 to 71 months. Observed prevalence of ECC was 41.1%. Although no statistically significant difference was found, children with ECC compared to healthy children were living in parts of the country with averages of ≈122€ lower GDP per capita, ≈4€ lower social and health care expenditures per capita, 9 inhabitants per km^2^ lower population density, almost 7€ per capita lower local self-government budget and a 0.6% higher unemployment rate. Furthermore, although without a statistically significant difference, untreated ECC was associated with ≈302€ lower GDP per capita, ≈12€ lower social and health care expenditures per capita, 34 inhabitants per km^2^ lower population density, almost 20€ per capita lower local self-government budget and a 1.7% higher unemployment rate.

**Conclusions:** This study, performed in a nationally representative sample of preschool children, revealed the association of economic macro-level factors with ECC prevalence and its (non-) treatment. Further research on a larger sample is necessary to confirm the results. These findings suggest that most of the public-health efforts regarding prevention and early treatment of ECC should be directed at regions with lower economic performance.

## Introduction

In the era of worldwide epidemic of non-communicable diseases (NCDs) (1), and considering oral disease as being part of this group and sharing the same etiology (2), poor oral health in preschool children has arisen as a public health issue that needs attention (3). In order to reduce disease burden, public health experts recognized the necessity of introducing a feasible prevention program that would be in accordance with population needs—therefore, understanding the etiological concept of oral disease as NCD might represent the crucial part of this process.

The current concept of the etiology of childhood caries suggests a strong impact of micro-level factors described in two groups: *risk factors* (social/behavioral, clinical) and *protective factors* (exposure to fluoridated water, utilization of fluoridated toothpaste and regular dental services) (4). However, if considering oral disease as NCD, the process of prevention planning needs to involve introduction and understanding of other factors that strongly correlate with health inequalities and originate from macro- or country-level factors such as social, economic, environmental and political determinants (5). Poor health is strongly connected to low productivity, country market value and economy performance, and it also affects the welfare of inhabitants (6). However, little is known regarding the social determinants of oral health in children and youth (7). Development and implementation of appropriate preventive strategies and adequate policies requires thorough oral health knowledge from a social and economic point of view (8).

Although Serbia has universal health coverage (UHC) for pediatric dental care and a similar distribution of physicians and dentists per 1,000 inhabitants, results from previous studies showed high prevalence and wide variation of ECC within the country (8). Having this in mind, the main goal of this study was to elucidate the possibility of an association between economic and healthcare macro-level factors and ECC profile in Serbian children.

## Method

### Study Design

This was an ecological study using data published in the statistical yearbook of municipalities, cities and regions in the Republic of Serbia (9) and data derived from a pathfinder cross-sectional survey with a nationally representative sample of preschoolers in Serbia.

Informed and written consent was obtained from parents before the children's examinations. Furthermore, the appropriate approvals from the following authorities were obtained: Ethical Committee of University of Belgrade School of Dental Medicine (approval number 36/10), Ministry of Health Republic of Serbia, management of primary health care centers (Head of the Staff) and management of kindergartens.

### Setting

By the Law of the Regional Development and the Law of the Official Statistics, Republic of Serbia is divided into 4 statistical territorial units: (1) Sumadija (central) and Western Serbia, (2) Southern and Eastern Serbia, (3) the northern region called Vojvodina, and (4) the City of Belgrade (the capital) (10). Each statistical territorial unit is a geographic entity and corresponds to the administrative division of the country. This subdivision of the country was made in accordance with European Union (EU) principles and methodology—the Serbian national nomenclature of statistical territorial units has been harmonized with the European nomenclature of territorial units for statistics (NUTS), and they correspond NUTS level 2 (regions of the country) (11). These statistical territorial units are used for purposes of measuring regional economic performance, gathering data, statistical analysis, social and health care planning, etc.

### Participants and Sampling Method

The study sample consisted of children aged 36 to 71 months of age attending public kindergartens in Serbia. As suggested by the World Health Organization's (WHO) Basic Methods for Oral Health Surveys, stratified cluster sampling was used in order to obtain a nationally representative sample: additionally, it was stratified according to three types of residency (urban, peri-urban and rural) involving most important population subgroups with possible differences in the disease level (12). Therefore, overall prevalence might be considered nationally representative. The WHO suggests at least 12 randomly chosen locations (600 participants) to obtain a representative sample (12). Out of 158 primary health care center locations in the whole country (6), 17 locations were randomly chosen to obtain an adequate distribution of data regarding urban, peri-urban and rural areas in each analyzed statistical territorial unit—we aimed to involve at least 850 participants (Table 1). Additionally, in each location—adequate urban/peri-urban/rural sites—public kindergartens were randomly chosen with the approval of local authorities. The study participants from each site (kindergarten) involved children attending randomly chosen kindergarten groups.

**Table 1:** Schematic equation of sampling method.

4 sites in the capital (2 urban and 2 peri-urban) (4 × 50 = 200) 2 sites in 3 large towns (1 urban and 1 peri-urban in each town) (6 × 50 = 300) 1 urban site in 3 large towns (urban) (3 × 50 = 150) 1 site in 4 rural areas (4 × 50 = 200)
Total = 17 sites × 50 = 850

### Variables

The oral health outcome variables for this study were:

- **ECC prevalence in the 36- to 71-month age group** calculated as total number of decayed, filled or missing teeth due to caries.
- **Frequency of untreated ECC in 36 to 71 months age group** calculated as total number of teeth with caries lesions, fillings with secondary caries and caries sequels.

Independent variables included data on macro-level factors used or calculated from the documents available at the official web page of the Statistical Office of the Republic of Serbia and involved country information on economic performance, population characteristics and healthcare system factors (10):

- **Regional gross domestic product (GDP) [per capita, 1,000 Serbian dinars (RSD)]**—represents the sum of value added of all local units that are active in the territory of the subject region (plus taxes on products less subsidies on products). Regional GDP “is the main indicator used for measuring the regional economic performances and the effectiveness of the regional policies and programs aimed at reducing the gaps between the regions in socio-economic development and distribution of the national wealth” (10);
- **Social and health care budget beneficiaries' expenditures (per capita, 1,000 RSD)**—involved data regarding budget beneficiaries' expenditures on human health and social work activities. These data were collected via the national regular statistical annual survey on budget beneficiaries and balance of payment received from the treasury administration, and published in the statistical yearbook of municipalities, cities and regions (9);
- **Local self-government budget (per capita, RSD)**—involved data on total budgetary revenues in each statistical territorial unit of the country covering all budgetary beneficiaries that are financed from the local government funds, consisting of current (tax and non-tax) revenues, receipts from selling non-financial assets and receipts from borrowing and selling financial assets. These data are collected and processed by the Ministry of Finance and published in the statistical yearbook of municipalities, cities and regions (9);
- **Unemployment rate (%)**—was calculated by subtracting the registered number of employed persons (persons who have contract of employment for a limited or unlimited time period, persons who are self-employed and run their own business and those who take agricultural activities) from the working age population (15–64 years) and presented as a percentage (10);
- **Population density (n)**—number of inhabitants in each statistical territorial unit of the country per 1 km^2^ of area; the data that were used involved population estimates for the post-census 2017 according to the population estimate as of the end of the previous year (December 31st, 2016) in addition to data on the results of processed statistics on natural and mechanical migrations of the population in 2017, published in the statistical yearbook of municipalities, cities and regions (9);
- **Density of physicians/dentists (n)**—number of inhabitants per one physician/dentist in each statistical territorial unit of the country: these data are in accordance with the Institute of Public Health of Serbia “Dr. Milan Jovanovic Batut” and are gathered from health care institutions in the Republic of Serbia, which are included in the Plan of Health Institutions Network, published in the statistical yearbook of municipalities, cities and regions (9).

### Data Sources

The Oral Health Assessment Form for Children according to teeth surfaces (12) was used for collecting data. All oral examinations were done from March to December 2018, in the field (in kindergartens) by calibrated primary health care pediatric dentists, using the plain dental mirror to detect caries lesions, gauze for drying the tooth surface and natural light with positioning the subject to get the best possible illumination (12).

### Bias

The calibration workshop was performed prior to data collection, involved all the examiners and included analyzing patients' photos with different stages of caries lesions on primary teeth. The examiners were instructed to detect any cavitated or non-cavitated lesions on the surface of primary teeth (13).

### Study Size

The sample size calculation used for this study included proposed at least 50 participants per location or at least 600 participants in a sample (Table 1). A sample size of at least 50 participants per location was chosen since the results from previous epidemiological studies in Serbia determined caries prevalence in more than 80% of children in the referent age group (12-year-olds) (12, 14–19).

### Statistical Methods

Results were presented as frequencies (percentages) or mean ± standard deviation (SD) depending on data type. The differences in the means of variables were analyzed using the independent *t*-test in case of normally distributed continuous variables or Mann–Whitney *U* test for non-normally distributed continuous variables. A probability value of <0.05 was considered significant. SPSS version 20 (SPSS Inc, Chicago, IL) was used for the statistical analysis.

## Results

The final sample included a total of 864 children aged 36–71 months. ECC was present in 355 (41.1%) examined children. Of these, untreated ECC was present in 326 (91.8%) children. Caries prevalence was statistically significantly (*p* < 0.01) higher in rural and peri-urban locations (44.9 and 46.5%, respectively) compared to urban areas (36.3%).

Physician and dentist density were similar across all country territorial units. Although no statistically significant associations were observed between oral health outcome variables and healthcare professionals' density, the results showed that children with untreated ECC compared to children with treated caries lived in the parts of the country with almost 14 inhabitants more per physician and almost 29 inhabitants more per dentist (Tables 2, 3).

**Table 2:** Regional social economic indicators regarding ECC in primary teeth.

	**Participants with healthy teeth (mean ± SD)**	**Participants with ECC (mean ± SD)**	**Total (mean ± SD)**	***p***
GDP per capita (1,000 RSD)	601.6 ± 245.6	587.2 ± 241.3	595.7 ± 243.8	0.4
Physician density (# inhabitants per 1 physician)	361.3 ± 46.9	363.3 ± 45.8	362.1 ± 46.5	0.9
Dentist density (# inhabitants per 1 dentist)	4442.2 ± 298.8	4444.2 ± 288.1	4442.9 ± 294.3	0.9
Social and health care expenditures per capita (1,000 RSD)	27.3 ± 6.4	26.8 ± 6.3	27.1 ± 6.4	0.2
Population density (# inhabitants per km^2^)	188.6 ± 195.6	179.9 ± 190.9	185.0 ± 193.6	0.4
Unemployment rate (%)	57.8 ± 10.5	58.4 ± 10.3	58.1 ± 10.4	0.4
Local self-government budget per capita (RSD)	39857.2 ± 12892.2	39064.6 ± 12969.4	39531.6 ± 12810.7	0.2

**Table 3:** Regional social economic indicators regarding untreated ECC.

	**Participants with treated ECC (mean ± SD)**	**Participants with untreated ECC (mean ± SD)**	**Total (mean ± SD)**	***p***
GDP per capita (1,000 RSD)	619.9 ± 269.8	584.3 ± 238.8	587.2 ± 241.3	0.9
Physician density (# inhabitants per 1 physician)	350.6 ± 48.4	364.4 ± 45.4	363.3 ± 45.8	0.1
Dentist density (# inhabitants per 1 dentist)	4417.2 ± 372.2	4446.6 ± 279.9	4444.2 ± 288.1	0.9
Social and health care expenditures per capita (1,000 RSD)	28.1 ± 6.8	26.7 ± 6.3	26.8 ± 6.3	0.3
Population density (# inhabitants per km^2^)	211.2 ± 212.3	177.1 ± 188.9	179.9 ± 190.9	0.9
Unemployment rate (%)	56.8 ± 11.4	58.5 ± 10.1	58.4 ± 10.3	0.9
Local self-government budget per capita (RSD)	41258.1 ± 13887.1	388869.8 ± 12589.7	39064.7 ± 12696.5	0.3

Although no statistical significance was found, the analysis showed that ECC-affected children, compared to caries-free children, were living in parts of the country with an average of 14400RSD (≈122€) lower GDP per capita, 500RSD (≈4€) lower social and health care expenditures per capita, 9 inhabitants per km^2^ lower population density, almost 793RSD (≈7€) lower local self-government budget per capita and a 0.6% higher unemployment rate (Table 2).

Further analysis showed children with untreated ECC compared to children with treated ECC were more likely to live in parts of the country with an average of 35600RSD (≈302€) lower GDP per capita, 1400RSD (≈12€) lower social and health care expenditures per capita, 34 inhabitants per km^2^ lower population density, almost 2390 RSD (≈20€) lower local self-government budget per capita and a 1.7% higher unemployment rate (Table 3).

## Discussion

This is the first study to determine the prevalence of ECC in preschoolers aged 36–71 months in the Republic of Serbia using a nationally representative sample of children, in accordance with the methodology proposed by the World Health Organization (12). The established ECC experience in a nationally representative sample of Serbian children aged 36–71 months was 41.1%, which is alarmingly high, considering that Serbia has pediatric UHC for dental and general healthcare and a similar density of physicians and dentists in each national territorial unit.

According to our knowledge, this is the first study to analyze the connection between macro-level social economic indicators and prevalence of ECC on a country level. Furthermore, macro-economic analysis showed that, although no statistically significant difference was found, oral health outcomes were associated with country economic performance: higher ECC prevalence and untreated ECC were more frequently observed in parts of the country with lower GDP per capita, lower social and health care expenditures per capita, lower population density, lower local self-government budget per capita and higher unemployment rate.

Although our study provided novel information on the association of ECC and country economic performance, the caution is necessary when interpreting the results due to some limitations.

Firstly, the obvious limitation could be the calibration procedure—the training of all examiners on patients was not feasible since the ethical committee and parental associations in the kindergartens would not approve participation of young children in the exhausting calibration process. In order to avoid possible non-consistency, the project team leader and peers reviewed all the answers so consensus during examination could be ensured.

Secondly, another possible limitation of our study could be the fact that all examinations were performed in the field (in kindergartens). However, although this method might potentially result in overlooking some of the non-cavitated caries lesions, this method is endorsed as the most practical and suitable for epidemiological studies in nationally representative samples (12).

Thirdly, the possible limitation, especially considering risk assessment, would be using the dmft (DMFT) index instead of ICDAS; therefore, the authors acknowledge possibly underreporting ECC prevalence since some non-cavitated lesions might be overlooked. Although ICDAS allows more detailed recording on non-cavitated lesions, dmft records both past and present ECC experience as well as (non-) treatment (20). Furthermore, it has been known and used for years by the primary health care dentists who were calibrated examiners for our survey. Although the WHO's Basic Methods for Oral Health Surveys (12) suggested excluding early stages of caries (white spots) because of the difficulty in distinguishing them in epidemiological field examinations without dental units, in order to have the most extensive possible coverage and the results representative of the population, the examiners were instructed to note if any non-cavitated lesions were observed during the survey.

Furthermore, since the data on the frequency of sugar consumption in different statistical territorial units of the country were not available, they were not used as a confounder for this study. This could be considered as a study limitation.

Finally, the possibilities of ecological fallacies should be highlighted as a possible limitation of the study. Furthermore, considering that this is a cross-sectional study, cause-effect relationships could not be assessed.

El Tantawi et al. (7) published in 2018 the first study that highlighted the connection between ECC prevalence and different health care and economic determinants of the United Nation's 193 countries—better ECC data availability and lower ECC prevalence was observed in countries with UHC. A high prevalence of ECC in preschoolers suggests an obvious presence of a gap in oral health care utilization.

However, despite pediatric general and dental UHC (21) and similar distribution of physicians and dentists across the country (10), the present survey showed a high prevalence of poor oral health in Serbian children −41.1% of children aged 36 to 71 months had ECC. Even more alarming is the fact that despite two National Oral Health Prevention Programs (1996–2000, 2009–2015) which raised concerns about oral health of Serbian children (22), we are observing the worsening of children's oral health during last 10 years when compared with the results of the oral health survey from 2009 conducted according to the same method (30.3%) (8).

Number of dentists and physicians in a region represent strong indicators of availability and accessibility of primary oral health care in preschool children (7, 23). Having in mind the high prevalence of oral disease in the population and the obvious lack of professionals (14), we could assume that most commonly provided treatments could be urgent, also leading to the UHC utilization gap. However, this should be confirmed in future studies.

In having a clear understanding of ECC as an NCD as well as identifying the national prevalence and differences in the disease level and risk subgroups, we fulfilled the essential requirements for designing effective preventive intervention according to population needs (24–26). Our results suggested influence of social determinants on ECC, therefore emphasizing the need for properly understanding and treating preschoolers' oral health (as NCD) using preventive strategies based on carefully planned policies. Identifying high-risk children is a task that requires special attention (27). Knowing that people from lower social economic levels rarely use dental health care (28), and having in mind that our data indicated higher proportion of children with ECC in regions with worse economic performance, it is necessary to confirm this hypothesis with a larger sample of children. Then, it would be possible to design a proper and cost effective preventive strategy that targets most vulnerable risk groups—the youngest possible and children with lower socio-economic profile.

The results of the present study suggested that health is much more than “*medical/dental knowledge and technologies”*—it involves social, economic indicators and polices (5). Social injustice and economic inequalities lead to worsening of health in people with lower socioeconomic position—these health inequities are unjustifiable and are avoidable (29). Every child—no matter of place of birth, living conditions, social or economic position, deserves to develop his or her full potential for good oral health. Our results suggest that country economic performance might be considered as a strong indicator of oral health.

## Data Availability Statement

The datasets generated for this study are available on request to the corresponding author.

## Author Contributions

AV conceptualized and designed the study, developed study methods, collected data on macro level factors, planned analysis, conducted the analysis and interpretation of data, drafted the initial manuscript, and reviewed and revised the manuscript. DM designed the study, interpreted the data, and critically reviewed and revised the manuscript. RV, TP, and IS contributed to the collection of macro-level and disease prevalence data, were involved with the data analyses and reviewed and revised the manuscript for important intellectual content. GC contributed to study methods, data collection and data analysis, and critically reviewed and revised the manuscript. All authors approved the final version of the manuscript.

### Conflict of Interest

The authors declare that the research was conducted in the absence of any commercial or financial relationships that could be construed as a potential conflict of interest.
